# System design and production practices of aquaponic stakeholders

**DOI:** 10.1371/journal.pone.0266475

**Published:** 2022-04-01

**Authors:** D. Allen Pattillo, Janelle V. Hager, David J. Cline, Luke A. Roy, Terrill R. Hanson

**Affiliations:** 1 Environment, Natural Resources, and Sea Grant Extension, University of Maryland, Queenstown, MD, United States of America; 2 School of Aquaculture and Aquatic Sciences, Kentucky State University, Frankfort, KY, United States of America; 3 School of Fisheries, Aquaculture, and Aquatic Sciences, Auburn University, Auburn, AL, United States of America; University of California Davis, UNITED STATES

## Abstract

Aquaponics is an agricultural practice incorporating aquaculture and hydroponic principles. This study assesses the current system design and production practices of the aquaponic industry, compares these metrics by stakeholder group, identifies trends, and provides recommendations for future development. An electronic survey of aquaponic stakeholders was conducted from December 2019 to June 2020 targeting hobbyists, producers, and educators from various aquaponic-focused professional associations, email and social media groups. Of 378 total responses, 84% came from the United States and were clustered in plant hardiness zones five to nine. Aquaponic systems were commonly homemade/do-it-yourself (DIY), many of which incorporated commercially available (turn-key) technology. Most growers used coupled systems that integrated recirculating aquaculture systems and either deep-water culture (DWC) or media bed hydroponic units. Common plant lighting sources were sunlight and light emitting diode (LED). Water sources were typically municipal or wells. Personal labor input was typically less than 20 hrs/wk. Funding sources were primarily personal funds, followed by government grants, and private investor funds. System sizes varied greatly, but the median area was 50 to 500 ft^2^ for hobbyists and educators and 500 to 3,000 ft^2^ for producers. Respondents commonly sold vegetable produce, training and education, food fish, and microgreens. Tilapia and ornamental fish were commonly grown, with 16 other species reported. Common crops were lettuce, leafy greens, basil, tomatoes, peppers, and herbs with many additional lesser-grown crops reported, including cannabis. Overall, the industry still growing, with a large portion of stakeholders having less than two years of experience. However, veteran growers have remained in operation, particularly in the producer and educator groups. The survey results suggest a shift away from outdoor systems, media beds, tomatoes, ornamental fish, and perch production, and a shift toward decoupled systems, DWC, drip irrigation, and wicking beds, larger system area, leafy greens, and trout/salmon production compared to previous industry surveys. The reduced diversity of plant species grown suggest some level of crop standardization. Commercial producers tended to sell more types of products than other stakeholders, suggesting that diversification of offerings may be key to profitability. The combined production area specified by respondents indicates the industry has grown substantially in recent years. Finally, the presence of bank loan-funded operations suggests increased knowledge and comfort with aquaponics among lenders.

## Introduction

Aquaponics is an agricultural practice incorporating aquaculture and hydroponic principles. Feeding fish generates nutrient-rich effluent used to fertilize plant crops, preventing its release into the environment [1]. Many practitioners are attracted to aquaponics for its resource efficiency, environmental benefits, and ability to produce healthy foods locally [2]. Although various forms of aquaponics have been practiced for centuries [3], modern aquaponic research began in the late 1970’s and has spread globally [4, 5], with much of the activity being in the United States (U.S), Canada, Europe, and Australia [6].

The aquaponic industry is growing rapidly. Guidance on best practices from credible sources is needed to usher in new growers. Previous industry surveys [6–15] and metanalyses [16, 17] have established some of the baseline conditions, practices, and trends for research and production. However, in this fast-growing industry, it is important to periodically document current practices and factors that would affect one’s decision to start an aquaponics business to insure end-user success [18–20]. The literature suggests that there are three major types of aquaponic practitioners–backyard hobbyists, commercial producers, and educators at all levels [6–8, 14]. These groups have different goals and needs, which impact their facility infrastructure, inputs, and practices. The goal of this survey was to expand on previous aquaponic industry knowledge, providing updated information on stakeholder-specific background and experiences, production systems, practices used, facility scale, production inputs, and crops produced. This research identifies industry trends that are relevant for hobbyists, producers, educators, and other aquaponic industry supporting groups.

## Materials and methods

An industry-wide online survey (Qualtrics XM, Provo, UT, USA) was conducted with the purpose of assessing the production practices of aquaponic stakeholders. The survey tool [21] contains a combination of original questions and topics synthesized from prior industry surveys [6, 15]. Responses were collected using a mixture of question formats to obtain qualitative and quantitative data. The survey format and question clarity were validated by beta version within the Aquaponics Association membership prior to Institutional Review Board approval (IRB Protocol No: 19–544 EX 1912). Participation was garnered from aquaculture and aquaponic listservs and social networking platforms. A snowball survey advertising method was used to encourage greater participation beyond the reach of our network [6, 22, 23]. Data collection spanned from December 10, 2019 to June 4, 2020 (177 days). The full survey and dataset are open access [21].

Respondents differentiated themselves by selecting a discrete stakeholder group–hobbyist, producer, or educator. All groups received the introductory block (21 questions), training/work hours (2), fish production (11), plant production (14), food safety (7), demographics (8) and a wrap-up block to collect voluntary survey feedback and contact information (4). Educators received 10 classroom usage questions and producers received 23 additional questions about business and marketing. The survey results are reported based on available responses. The survey duration was expected to be 20 minutes or more depending on the stakeholder group, although the survey length and depth of questions likely extended this duration and may have led to dropout. This anonymous survey was conducted in English and in an online format, which likely limited our response pool compared to previous surveys [6] and primarily represent responses from the United States.

### Statistical analysis

Response data was reviewed by investigators to eliminate incomplete responses. Extreme outliers were identified using SPSS Statistic 26 (IBM, Armonk, NY, USA) and removed from analysis. Figures were generated using Excel (Microsoft 360, Redmond, WA, USA). Maps were generated using Mapchart.net, licensed under CC BY 4.0. The number of potential respondents is unknown when using social media platforms, thus a reliable response rate could not be calculated. The number of total responses per question (N) and stakeholder group responses (n) per question varied and is noted in each table and figure. The central tendency and spread of the data are expressed using descriptive statistics (e.g. mean ± standard deviation (SD)) and responses were generalized using proportions (e.g. percentage). Continuous data were evaluated for differences among groups using a one-way Analysis of Variance (ANOVA) test with Tukey’s Post-Hoc test for pairwise comparisons (α = 0.05). Where appropriate, data was transformed with the natural logarithm function to meet normality assumptions. Spearman’s non-parametric correlation coefficient (ρ) was used to measure the strength and nature of relationships between variables using the Bonferroni adjustment for multiple comparisons (α = 0.05/n) to reduce the risk of Type I error [24].

## Results and discussion

### Demographics, background and experiences

**Fig 1:**
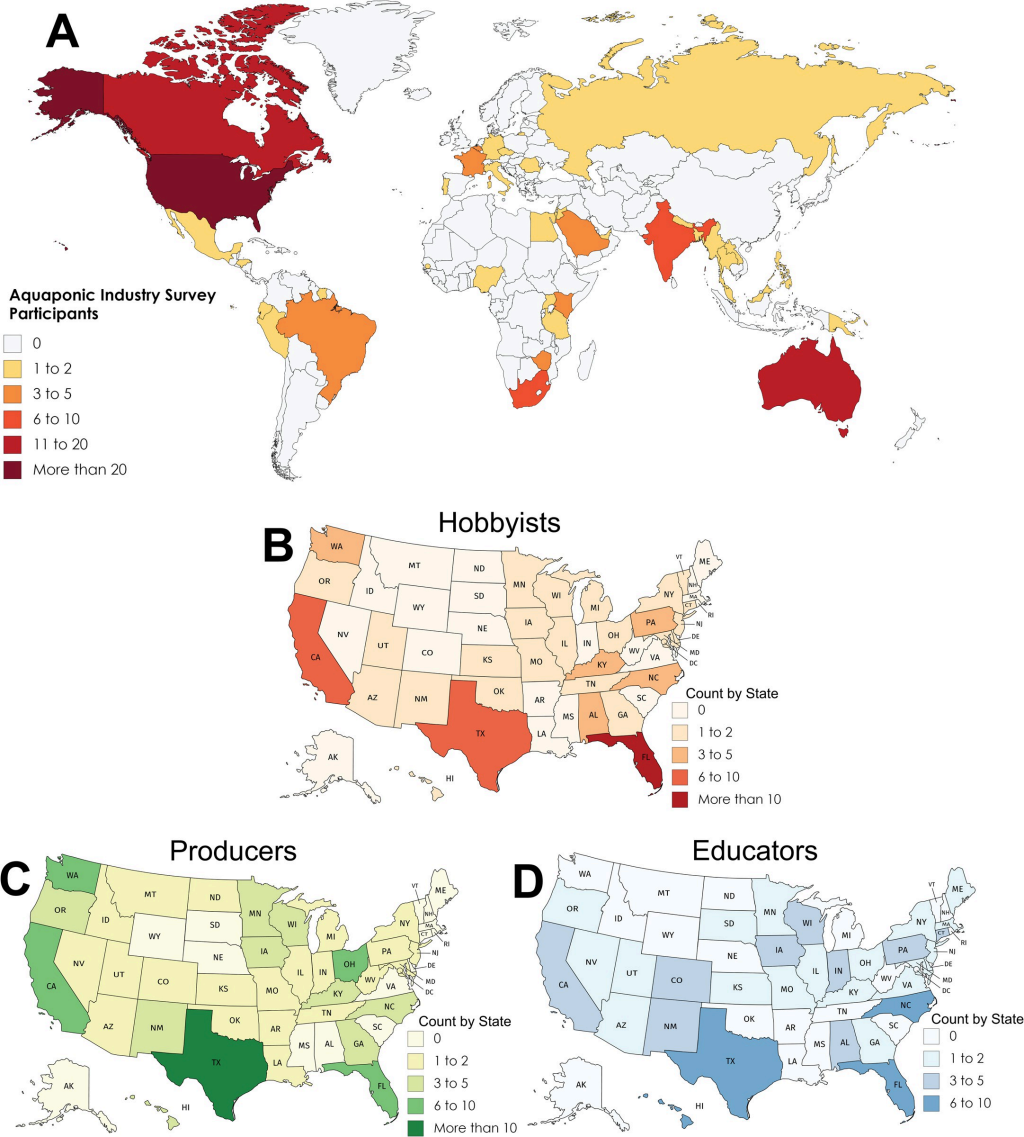
Geographical distribution of aquaponic industry survey participants. Maps show the number of respondents by country (A), and hobbyists (B), producers (C), and educators (D) by state within the United States. Maps were generated using Mapchart.net, licensed under CC BY 4.0.

Survey responses were collected from 378 individuals. The majority of participants were producers (41%) with educators and hobbyists representing 31% and 28%, respectively. This is a shift from 84% hobbyists, 57% educators, and 32% producers reported by Love et al. [6], although their stakeholder group delineations were not mutually exclusive. The median respondent age was 55 to 64 (hobbyist) or 45 to 55-years (producers and educators). Across all groups, most responses were from white (75%), men (80%), in the United States (84%; Fig 1), that achieved a bachelor’s degree or higher (71%). Educators were the most diverse group with 37% of respondents from other ethnic groups and 24% being women. Hobbyists were primarily retired (35%) or working full time (50%); whereas the majority of producers and educators were working full-time, 64% and 72% respectively. Respondents were at various stages of development in their operations from researching (15%) to planning (21%), constructing (7%), and currently operating (57%). All groups reported that most of their income came from sources other than aquaponic production.

**Fig 2:**
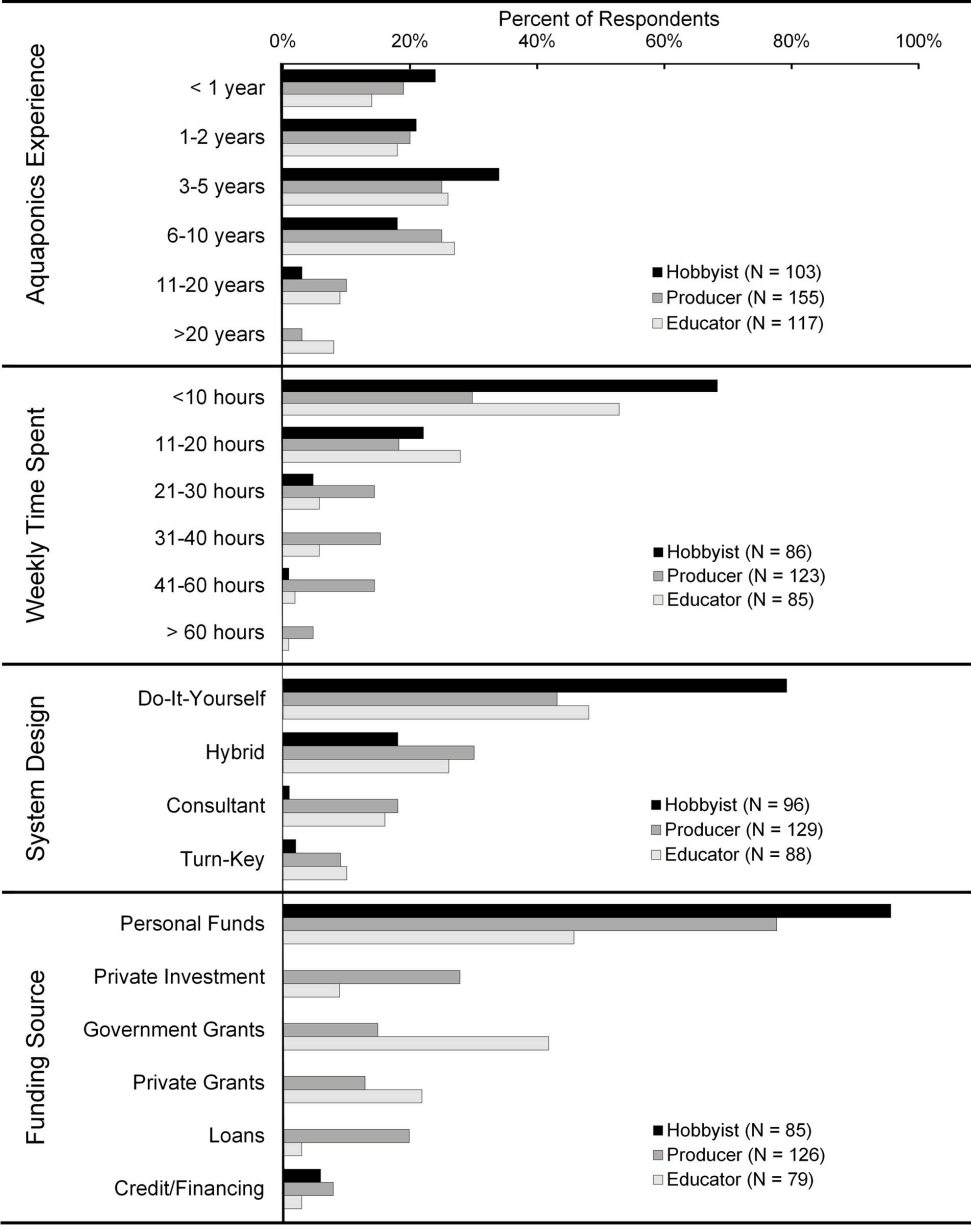
Survey respondent years of experience, weekly labor, system design, and funding sources.

The aquaponic experience level of the respondents overall was relatively low, with 19% having less than one year, 38% having less than 3 years, and 66% having five years or less (Fig 2). Love et al. [6] reported 89% having less than 5 years of experience, 52% with less than 3 years, and about 26% with one year or less. Love et al. [6] reported about 5% of respondents with more than 11 years of experience, compared to 11% of respondents in this study, suggesting some level of retention over time. Greater detail on demographic data is available from Pattillo [25].

### Location

**Fig 3:**
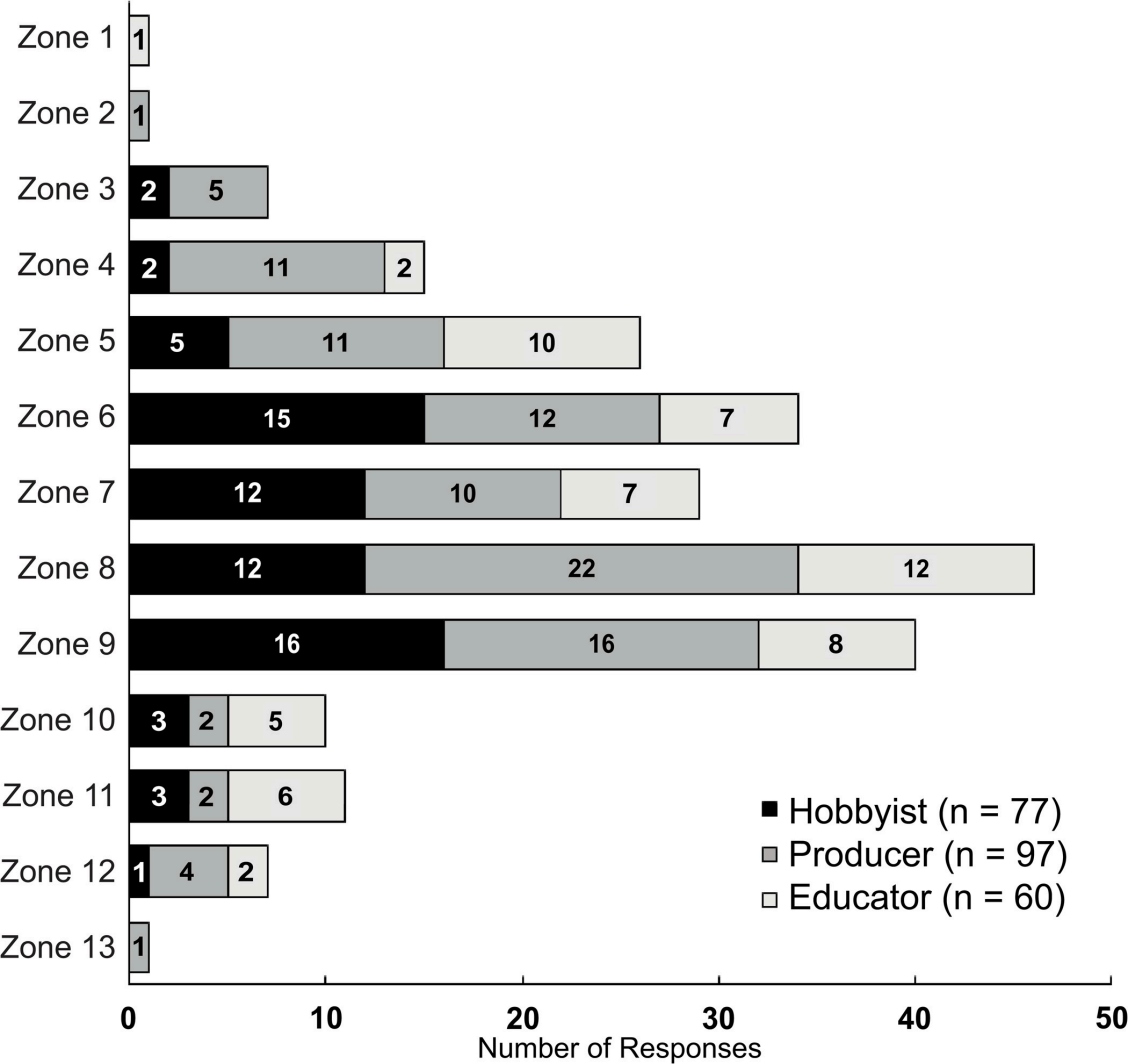
Number of U.S. survey respondents in each USDA plant hardiness zone. Zone 1 represents the most polar and zone 13 is the most tropical climates. Numbers preset within the bars represent the total number of respondents in a given zone for each stakeholder group.

Geography and climate impact what species of fish and plants can be easily grown, production strategies, input requirements, and environmental sustainability [26–29]. For example, in cold climates, Ghamkhar et al. [28] concluded that over 91% of global warming (CO_2_) and acidification (SO_4_) impacts of aquaponic operations are directly linked to generating heat and electricity. Additionally, locations with more stable environmental conditions encourage efficient nitrate uptake by plants and lower energy consumption [27]. In this study, location was assessed geographically, by plant hardiness zone, and by background setting. The geographical distribution of survey respondents is presented in Fig 1, which primarily represented individuals in the United States (n = 162). Seventy seven percent of operations (N = 228) were clustered in plant hardiness zones five through nine (Fig 3), encompassing the temperate and subtropical climate zones [30]. However, for those located in colder climates, farming in well-insulated buildings where the growing environment can be regulated is one cold weather adaptation that, when combined with vertical production, can maximize space and heating efficiency [29, 31].

Out of 344 responses, 45% were from rural areas, 27%, 26% and 2% from suburban, urban and industrial areas, respectively. Hobbyists were mostly located in rural (46%) and suburban (35%) settings. Texas had the most rural operations (n = 14), Florida had the most suburban operations (n = 8), and California had the most urban operations (n = 7). Producers were primarily located in rural (54%) and urban (26%) areas, while educators were evenly distributed across rural, suburban, and urban settings. Only six respondents operated in industrial settings, four of which were producers. Generally, rural environments have fewer zoning restrictions than urban areas, but access to inputs, infrastructure, and markets may be prohibitive [32]. Many view aquaponics in urban environments as an agricultural solution to food deserts (areas that lack access to grocery stores or healthy meal options). However, zoning restrictions and obtaining permits may be an issue for aquaponics in urban, suburban, and industrial environments. Appropriate tax incentives could make these under-utilized areas prime contenders for major aquaponic production sites, as they have the required infrastructure and proximity to markets. Alternatively, educators in rural, urban, and suburban setting are using aquaponic systems to teach Science, Technology, Engineering, Agriculture and Math (STEAM) topics [12, 14, 33].

### System design

System design options included homemade or do-it-yourself (DIY), commercially available (turnkey), a combination of homemade and commercially available (hybrid) or designed by a consultant. Out of 313 responses, 55% were DIY, 25% were hybrids, 12% were consultant designed, and 7% were turnkey (Fig 2). Hybrid and DIY categories together account for 80% of systems overall. Hobbyists almost exclusively used DIY and hybrid systems (Fig 2). Producers and educators used a wider variety of system designs; however, DIY and hybrid were most commonly reported. Approximately 10% of both producers and educators used turn-key systems (Fig 2).

Today, many of the commercial turn-key systems are too costly to provide an acceptable return on investment (ROI), which has led growers to develop their own systems. Eighty percent of aquaponic systems were DIY in this study, which is similar to the findings of Love et al. [6, 7] and Genello et al. [14]. The self-design of systems will likely continue, especially for hobbyists, and this market could be expanded with the development of benchtop and backyard system designs that are readily available and affordable. For producers, the market is likely shifting toward greater use of commercially available systems and components as the industry grows and matures, which is indicated by the elevated use of consultants and turn-key systems by producers and educators in this study.

### Coupling design

System coupling describes the water and nutrient flow dynamics of an aquaponic system. In coupled systems, water is recirculated between the fish and plant portions of the system in a continuous loop. In decoupled systems, water flows from the fish to the plants and does not return to the fish. Out of 325 responses, 84% used coupled systems, 13% used decoupled systems, and 3% were unsure. Hobbyists almost exclusively used coupled systems (92%) compared to 84% of producers and 85% of educators.

Coupled systems are the most common design due to the extensive amount of research, dating back to 1977, that has been conducted in the U.S., Australia, and Europe [34]. Coupled aquaponics is attractive to all user groups due to the established fish feed to plant ratios, proven system design, and documented economic information [35]. However, matching species physical and biological needs and tolerances is critical for a coupled aquaponic system [36] and may result in sub-optimal production of fish and plants if suitable ranges for each species do not overlap (e.g. Tilapia and lettuce).

In a decoupled system, aquaculture and hydroponic components can be separated, or decoupled, allowing for independent management of system parameters to optimize production in both components [37]. Strategies for decoupling include multi-loop recirculating systems [27, 38] and drain-to-waste irrigation systems [39, 40]. Additional strategies like drip irrigation of field crops using aquaponic effluent, characterized as ‘aquaponic farming’, may prove useful to farmers especially during the growing season [39, 41]. With additional research and acceptance, more growers may shift towards the decoupled design because it offers greater flexibility and control [42].

### Aquaculture unit

This section includes fish production methods, aquaculture system components, and production environment. Similar to Love et al. [6], the most common fish production method used by respondents (N = 334) was recirculating aquaculture systems (RAS) (70%), with lesser use of ponds (8%), raceways (e.g. flow through) (7%), cages (e.g. net pen) (5%), biofloc systems (4%) and other systems (6%) (Table 1). Hobbyists almost exclusively used RAS, while producers and educators used a wider variety of systems (Table 1).

**Table 1:** Aquaculture system components incorporated into aquaponic systems by hobbyists, producers, and educators.

System Components	Hobbyist (N = 72)		Producer (N = 102)		Educator (N = 62)	
	n	%	n	%	n	%
Water pump	67	93	97	95	56	90
Aeration	58	81	95	93	54	87
Clarifier/solids settler	38	53	70	69	30	48
Heater	35	49	57	56	26	42
Backup generator	15	21	66	65	29	47
Dedicated biological filter	18	25	60	59	25	40
Combination solids/biofilter	30	42	44	43	28	45
Environmental monitoring	10	14	43	42	17	27
Dedicated mechanical filter	15	21	35	34	17	27
Automated feeders	13	18	26	25	18	29
Airlift	13	18	25	25	11	18
Ultraviolet sterilization	10	14	28	27	10	16
Chiller	7	10	20	20	4	6
Pure Oxygen	3	4	18	18	1	2
Ozone sterilization	1	1	11	11	0	0
Protein skimmer	1	1	5	5	3	5
Production Method	(N = 79)		(N = 112)		(N = 66)	
Recirculating	73	92	103	92	59	89
Pond	6	8	11	10	10	15
Biofloc	1	1	9	8	3	5
Flow-through	2	3	15	13	5	8
Cage	0	0	10	9	7	11
Other	5	6	11	10	4	6
Growing Environment	(N = 77)		(N = 104)		(N = 65)	
Outdoors	18	23	22	21	15	23
Shade Structure	17	22	14	13	11	17
High Tunnel	4	5	11	11	2	3
Greenhouse	24	31	49	47	30	46
Indoors/Warehouse	24	31	36	35	25	38

Species needs vary greatly in terms of water quality, solids removal, temperature, disinfection, stocking density, harvesting and routine maintenance, impacting production cost and system design [43]. Respondents selected from the 16 recirculating aquaculture system components in Table 1 to describe their system. On average (± SD) producers used significantly more components (6.9 ± 2.9) than hobbyists (4.6 ± 2.3; *p* < 0.001) and educators (5.4 ± 2.5; *p* = 0.001). There was a range in system component usage, but most respondents used pumps and aeration, mechanical and biological filters, water heaters and backup generators. The cost of system components with respect to their benefit to growers can be interpreted by their frequency of use. Proportionally, hobbyists used backup generators, dedicated biological filters, environmental monitors, and protein skimmers less commonly than the other groups, but used aeration and automated feeders with similar frequency as other groups. Producers, who depend on their system operations to generate an income [7], used solids settling clarifiers, water heaters, backup generators, dedicated biological filters, environmental monitoring systems, chillers, pure oxygen, and ozone sterilization, more often than other groups, and used dedicated mechanical filters, airlift pumps and ultraviolet sterilization similar to other groups. Educators, who may conduct research or may not have time to tend to their systems [12], used heaters and chillers less often than other groups and used automated feeders more often than other groups.

The growing environment for fish impacts water temperature, nutrient concentrations due to precipitation or evaporation, algae growth, food safety and biosecurity. Outdoor environments are cost-effective, but offer no control over the elements, which limit the potential growing season. Alternately, indoor environments offer varying levels of environmental control but require energy to operate and can be costly to build. Overall (N = 246), the most common growing environments for fish production were greenhouses (32%) or indoors (27%), followed by outdoors (17%) or a shade structure (13%), with few using high tunnels (5%) (Table 1). This represents a shift away from outdoor production (47%) reported by Love et al. [6]. Producers and educators used greenhouses more frequently than hobbyists. Use of indoor and outdoor production environments was similar across groups. Hobbyists used shade structures more frequently than producers and educators. Producers used high tunnels more frequently than hobbyists and educators. On average, respondents used 1.3 ± 0.8 fish growing environments.

### Horticulture unit

This section describes plant growing environments [44], hydroponic systems [45], and lighting sources [46] that make up the horticulture unit [1]. Each of these components impact the types of plants that can be grown, the level of crop protection from pests and weather, startup cost and operational expenses. Greenhouses are among the most expensive production environments, but allow growers to control temperature, humidity, and light intensity as well as reduce pest and weather damage to crops [44, 47]. Warehouses are examples of indoor production environments and may be a viable option in colder climates, especially for out-of-season production when heating costs outweigh lighting costs and market prices are elevated [29]. Respondents (N = 223) indicated the most common plant growing environments were greenhouses (51%), followed by indoors (28%), and outdoors (25%), with fewer growers using shade structures (15%) and high tunnels (12%) (Table 2). Proportionally, about half as many growers use outdoor growing environments compared to previous years [6], indicating an investment in crop protection. Producers used greenhouses most frequently, while indoor growing environments were used more often by hobbyists and educators. Producers and hobbyists used cost-effective high tunnels more frequently than educators. “Other” growing environments included in-ground greenhouses (walipini), home basements, laboratories, and classrooms. On average, respondents used 1.3 ± 0.8 plant growing environments.

**Table 2:** Horticulture production system components for aquaponic hobbyists, producers, and educators.

Hydroponic Unit	Hobbyist (n = 68)		Producer (N = 94)		Educator (N = 57)	
	n	%	n	%	n	%
Deep Water Culture	39	57	75	80	42	74
Media Beds	53	78	51	54	36	63
Nutrient Film Technique	15	22	25	27	18	32
Drip Irrigation	11	16	20	21	10	18
Vertical Towers	14	21	18	19	11	19
Wicking Beds	13	19	16	17	8	14
Light Source	(N = 72)		(N = 93)		(N = 56)	
Sunlight	54	75	78	84	43	77
Incandescent	2	3	2	2	0	0
Fluorescent	20	28	17	18	12	21
High Pressure Sodium	5	7	5	5	2	4
Metal Halide	3	4	8	9	4	7
Light Emitting Diode	28	39	45	48	21	38
Induction	0	0	1	1	0	0
Growing Environment	(N = 72)		(N = 92)		(N = 59)	
Outdoors	21	29	20	22	15	25
Shade Structure/Canopy	14	19	11	12	8	14
High Tunnel	7	10	16	17	3	5
Greenhouse	26	36	57	62	30	51
Indoors	22	31	22	24	18	31

Our findings are similar to Love et al. [7] who reported that plant production was strictly either in a greenhouse (31%) or in a greenhouse in combination with other indoor and/or outdoor facilities (41%). Genello et al. [14] reported that educators grew plants outdoors (47%), in a greenhouse (46%), indoors (28%), or on rooftops (3%). Three quarters of hobbyist’s systems, however, were located at their home, either outdoors (50%), in a greenhouse (33%), or indoors (19%) [8].

Common types of hydroponic units include deep water culture (DWC or floating rafts), media beds (flood and drain or continuous flow), nutrient film technique (NFT), and drip irrigation (Dutch or Bato buckets and field crops), with growing interest in vertical tower production [29], wicking beds [48], and aeroponics [1, 6, 36, 45]. On average, respondents used 2.3 ± 1.3 plant production methods. Seventy one percent of respondents (N = 219) in this study used deep water culture systems (DWC) (e.g. floating rafts) and 64% used media beds (e.g. flood and drain). Fewer growers chose the nutrient film technique (NFT) (26%), vertical towers (20%), drip irrigation (e.g. Dutch or BATO buckets) (19%), or wicking beds (17%) (Table 2). Hobbyists used media beds most frequently, while producers and educators preferred DWC. Love et al. [6] reported the most common hydroponic methods were media beds (86%) and DWC (46%), with lesser usage of NFT (19%), vertical towers (17%), wicking beds (2%), or drip irrigation (2%). This indicates increased usage of DWC, drip irrigation, and wicking beds, while media bed usage decreased overall.

Media beds tend to be more common with hobbyists and educators who have smaller scale systems, this is likely related to their simplicity of design, cleaning requirements, and flexibility in production [8, 14]. Producers tend to use larger systems that incorporate DWC, which is lends itself to easy cleaning, crop mobility through the system, and flexibility of harvest [45]. Leafy greens are typically grown in DWC while vining crops tend to be grown in media beds [1]. The NFT method is also common, but presents management challenges, especially with clogging in the system [1]. Vertical production units, while space efficient, tend to have similar clogging and pump failure challenges [1]. Drip irrigation systems like Dutch buckets provide a modular production solution for vining crops like tomatoes [40] and cucumbers [49] and can also be adapted for outdoor soil crop production [39].

Plant lighting sources used by respondents (N = 221) were similar among groups, with sunlight (79%) being most common, followed by light emitting diode (LED) (43%), and fluorescent (22%) (Table 2). On average, hobbyists used 1.6 ± 0.7 light sources, while producers used 1.7 ± 0.9, and educators used 1.5 ± 0.7, which was similar across groups. The most common input for “other” light source was metal halide (n = 3, 1%). Hobbyists and educators primarily used sunlight, LED, and fluorescent lights. Producers relied most heavily on sunlight and LED lights.

Respondents were wise to take advantage of sunlight and energy efficient LED lighting. The expense of constructing a greenhouse environment and maintaining optimal light intensity, duration, and temperature for plant growth can be costly and logistically challenging. Sunlight is the ideal lighting source because it is free and provides heat. Indoor environments provide increased temperature control but are dependent on artificial light for plant growth and electrical usage can be seven times higher than in a greenhouse [29]. Studies comparing grow light technologies demonstrated that plants grown under LED lighting tend to achieve greater production biomass under the same conditions than other artificial lights and do so with lower energy consumption [46]. However, Nelson and Bugbee [50] found that the initial expense of obtaining the LED light fixtures compared to the industry standard high-pressure sodium (HPS) fixtures made the return on investment (ROI) of LED grow lights between 5 and 10 years. As LED technology advances, more cost-effective options may become available. Growers must compare these costs on a case-by-case basis to select the best and most cost-effective equipment for their situation.

### Facility size

**Fig 4:**
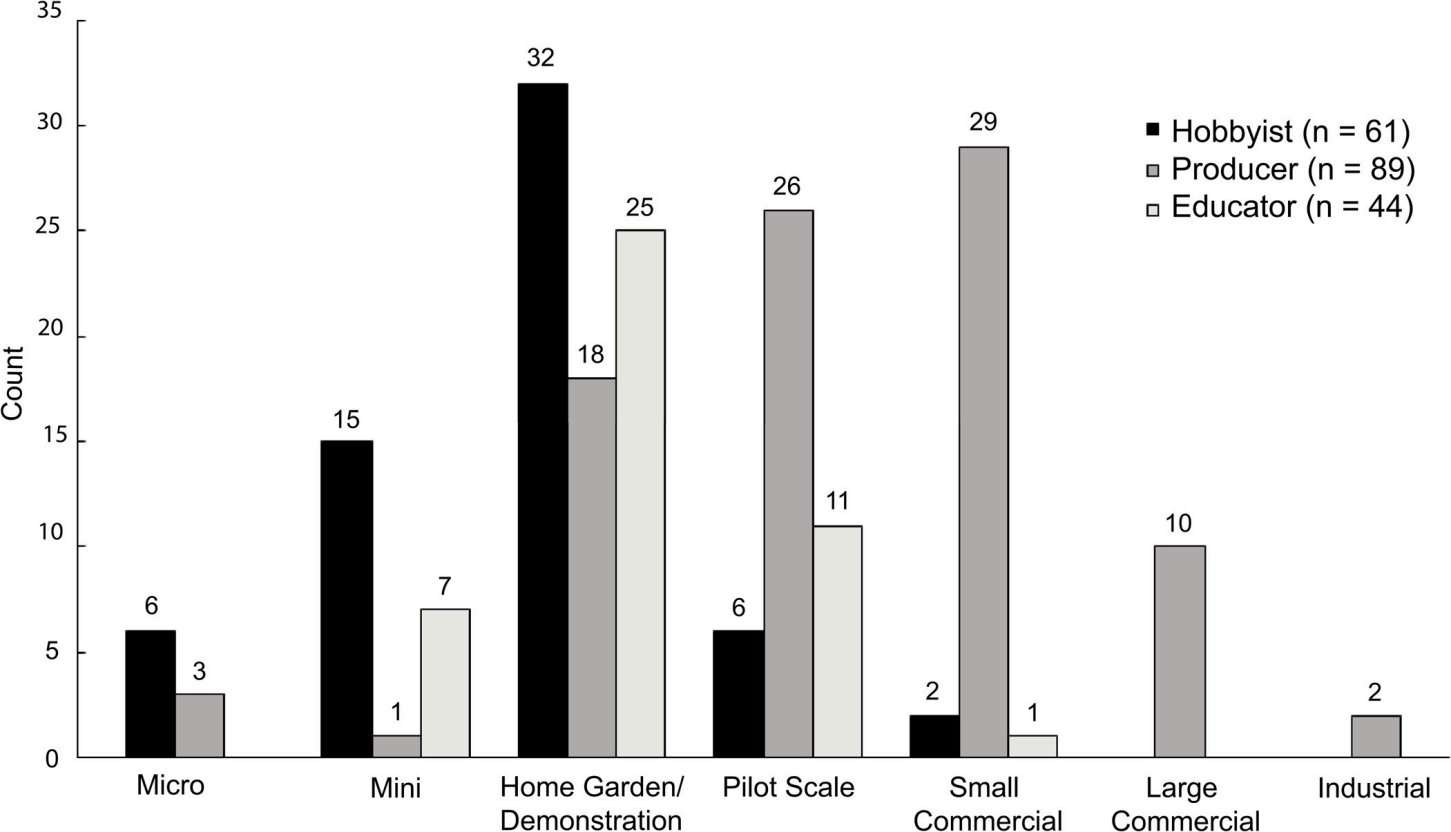
Size distribution of combined fish and plant area footprint of hobbyist, producer, and educator aquaponic systems. Numbers above bars represent the number of respondents. Size designations are ‘micro’ = < 10 ft^2^; ‘mini’ = 10 to 50 ft^2^; ‘home garden/demonstration’ = 50 to 500 ft^2^; ‘pilot scale’ = 500 to 3,000 ft^2^; ‘small commercial’ = 3,000 to 22,500 ft^2^; ‘large commercial’ = 22,500 to 165,000 ft^2^; ‘industrial’ = >165,000 ft^2^.

Production facility size relates to its output capacity, markets served, cost of production, and economic viability. The combined fish and plant area reported by all respondents (N = 204) totaled nearly 2.5 million ft^2^ and ranged from 6 ft^2^ to 871,200 ft^2^, with a median of 450 ft^2^ and an interquartile range from 100 ft^2^ to 3,200 ft^2^. Median facility size classes (Fig 4) were ‘home garden/demonstration’ (50 to 500 ft^2^) for hobbyists and educators and ‘pilot scale’ (500 to 3,000 ft^2^) for producers. Love et al. [6] reported a median facility footprint of 15 m^2^ (162 ft^2^) and range of 0.01 m^2^ (0.1ft^2^) to 18,580 m^2^ (199,993 ft^2^), indicating an increase in individual facility scale over time. Larger facilities tend to have lower per unit cost of production [51, 52]. König et al. [53] suggested that facilities need to be at least 1,000 m^2^ (10,764 ft^2^) to be profitable, which would encompass the area of approximately three and a half standard greenhouses. All 17 respondents meeting these criteria were producers, representing 19% of the producer group.

Hobbyists (44%), producers (76%), and educators (53%) all expressed an interest in scaling up their operations, indicating growth potential for the aquaponic industry. The need to scale up to reach an economically viable production level is a clear motivation for producers [7, 51, 52, 54]. However, motivations for deciding facility size by hobbyists may involve space availability, personal time, food needs, personal drive, and disposable income [6, 8]. Educators are more often motivated by the interest level of their students, availability of lesson plans, work time availability, support from their administration, and the availability of space and funding [12, 14].

### Labor inputs

Labor is a major consideration for any operation, particularly for businesses. The amount of personal time respondents (N = 294) spent working with their aquaponic system on a weekly basis is presented in Fig 3. About half of the respondents spent ≤10 hrs/wk and 23% spent 11 to 20 hrs/wk (Fig 3). The majority of hobbyists (71%) and educators (55%) spent ≤10 hrs/wk working on their systems and nearly all spent <20 hrs/wk. Producers tended to spend more time working with their systems, with 50% spending >20 hrs/wk and 20% spending > 40 hrs/wk. Weekly time spent was weakly positively correlated with years of experience (ρ = 0.272, *p* < 0.001) and very weakly correlated with development stage (ρ = 0.198, *p* = 0.001). Weekly time spent on system operation was moderately correlated with combined fish and plant production area (ρ = 0.532; *p* < 0.001). Producers devoted more time to their systems than hobbyists or educators, which aligns with the fact that their facilities tended to be much larger. Labor costs can be quite high for aquaponic producers, making up 49% of the total operating budget [52] and often determines their economic viability [26]. Larger facilities were shown to require more labor but development of automation, data modeling, and environmental sensing equipment to reduce labor and energy costs will be a major focus of future aquaponic innovation [18, 55, 56].

### Water source

The physical, chemical, and biological properties of the water source affects the productivity, nutrient dynamics [1, 57], and food safety [58, 59]. Out of 252 responses, the most commonly used water sources were municipal (47%) and groundwater wells (44%), followed by rainwater (26%), with very few using surface water (5%). Hobbyists (1.2 ± 0.4), producers (1.3 ± 0.5), and educators (1.4 ± 0.6) reported using multiple water sources per operation, which was similar among groups. Municipal and well water are the highest quality and readily available but chemical additives like chlorine or chloramine may harm fish and beneficial bacteria. Hobbyists and educators tended to use municipal water most often, while producers most commonly used well water. Rainwater was used more frequently by hobbyists and producers than educators. Producers used surface water most frequently. However, due to biosecurity and food safety issues, use of untreated rainwater and surface water is generally discouraged because they may harbor living organisms and pathogens [1, 58–60]. The relative use of municipal water and well water, followed by rainwater, with a few using surface water (e.g. ponds or streams) is similar to the findings of Love et al. [6].

### Funding source

Access to capital is a major barrier to entry for newcomers due to lack of access to bank loans for aquaponic farms [15, 20]. Producers often use their own personal funds or find private investors, while educators may be successful with obtaining government grants, obtaining donations, and selling education in addition to produce [14, 15]. In this study, out of 290 responses, 74% indicated that they use personal funds, followed by government grants (18%), and private investor funds (14%) (Fig 2). Hobbyists used 1.1 ± 0.2 funding sources, which was significantly lower than producers (1.7 ± 1.2) (*p* < 0.001) and educators (1.4 ± 0.7) (*p* = 0.021). Hobbyists were almost entirely self-funded (96%), while producers enhanced their personal funds (78%), with investor funds (28%) and bank or government loans (15%). Educators combined their personal funds (46%) with government grants (42%), and private grants (22%). Respondents often used more than one funding source in their operation, therefore the percentage of responses do not add to 100%.

Loan officers tend to be unfamiliar with aquaponics or have concerns about financial risk and the lack of viable business examples, which constrains financing options for growers. Due to the capital-intensive nature of commercial aquaponics, loan opportunities must be available for the industry to grow, yet these options will only exist when there is a low perceived risk to loan agencies [19]. To reduce risk to private lenders, government-backed loans could be made available, allowing farmers to get the financing they need [13, 61]. One such opportunity that could apply to aquaponic farmers is the federally funded beginning farmer and rancher loan program [62]. These programs have very specific qualification requirements for farmers, however. Interestingly, 9% of participants were able to secure loans, which is in contrast to previous surveys that reported no loan usage by participants [15]. This may indicate an increased awareness and comfort level with this new technology among financial institutions.

### Products sold

**Fig 5:**
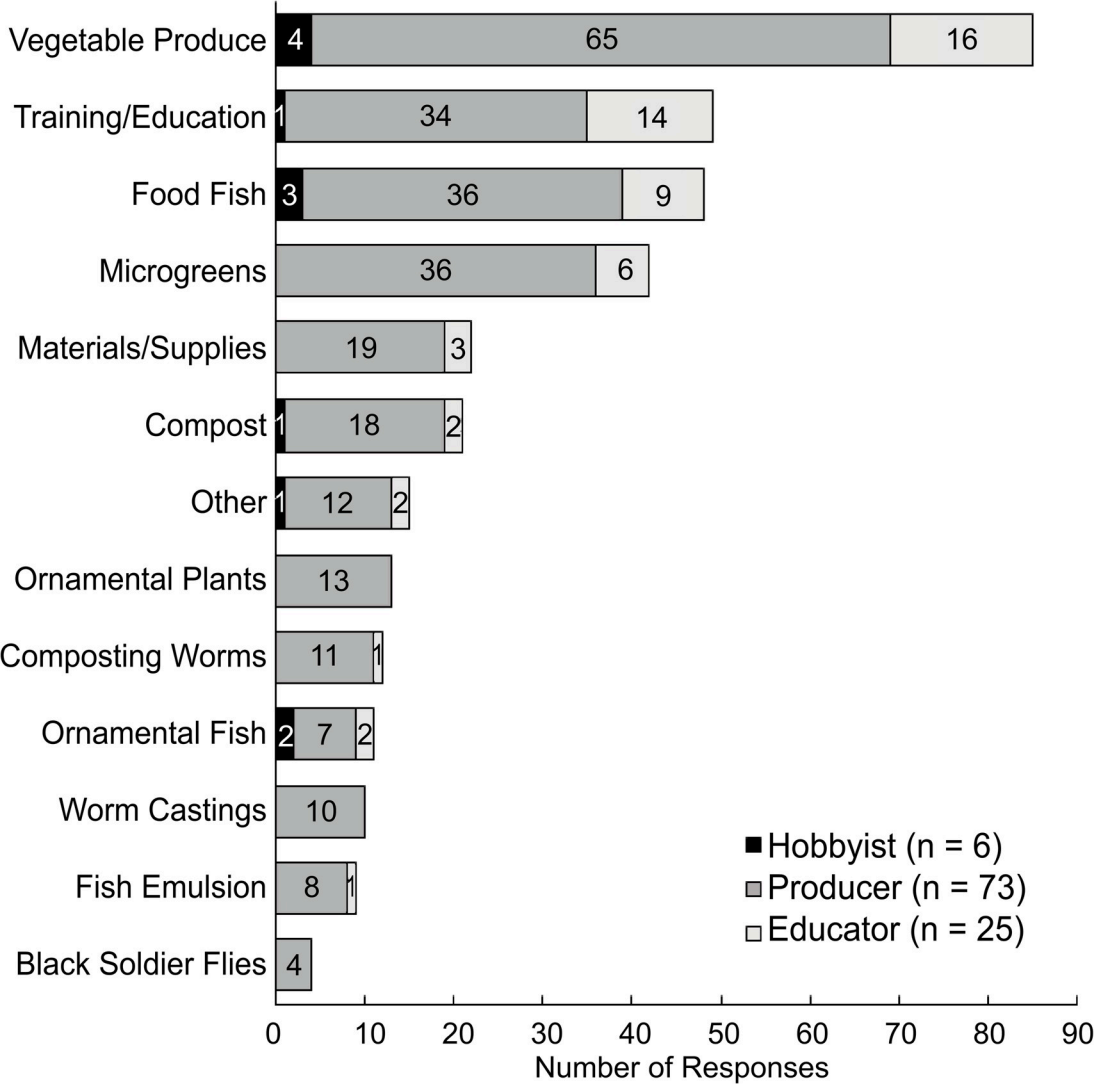
Frequency of product types sold by aquaponic hobbyists, producers, and educators. Numbers present within the bars represent the total number of selections made by each stakeholder group.

Aquaponic products were sold by 35% of respondents (N = 300), with only 57% currently in the production stage. The majority of hobbyists (93%) and educators (71%) did not sell products, but 57% of producers did. Hobbyists (n = 6) sold 2.0 ± 0.9 types of products per respondent, which was similar to educators (n = 24) who sold 2.3 ± 1.1 (*p* = 0.993). Producers (n = 73) sold 3.7 ± 2.2 types of products per respondent, which was significantly more than educators (*p* = 0.013) but not hobbyists (*p* = 0.194), due to a small sample size. Vegetable produce was by far the most common product sold, followed by training and education, food fish, and microgreens (Fig 5). To a lesser extent, materials, supplies, and compost were sold, with very few respondents selling ornamental plants, composting worms, ornamental fish, worm castings, fish emulsion, and black soldier flies. Hobbyists sold up to three products, including vegetable produce, food fish, and ornamental fish (Fig 5). Producers sold up to 12 product types, but sold vegetable produce, food fish, microgreens, and training and education most frequently. Educators sold up to five products, mostly consisting of vegetable produce, training, and education. Incorporation of agritourism, educational opportunities, and selling non-food products related to aquaponics is common practice to generate a profit [18]. Love et al. [7] reported that commercial producers sold fish and plants (37%), materials and supplies (27%) or some combination of both (36%), and 47% of aquaponic farmers conducted other farming enterprises. Villarroel et al. [15] found that only 12% of their respondents actually sold crops, while 24% sold materials and supplies, and 65% provided aquaponic training and education. Our results suggest that diversifying product offerings may be necessary for economic viability.

### Aquatic species produced

**Fig 6:**
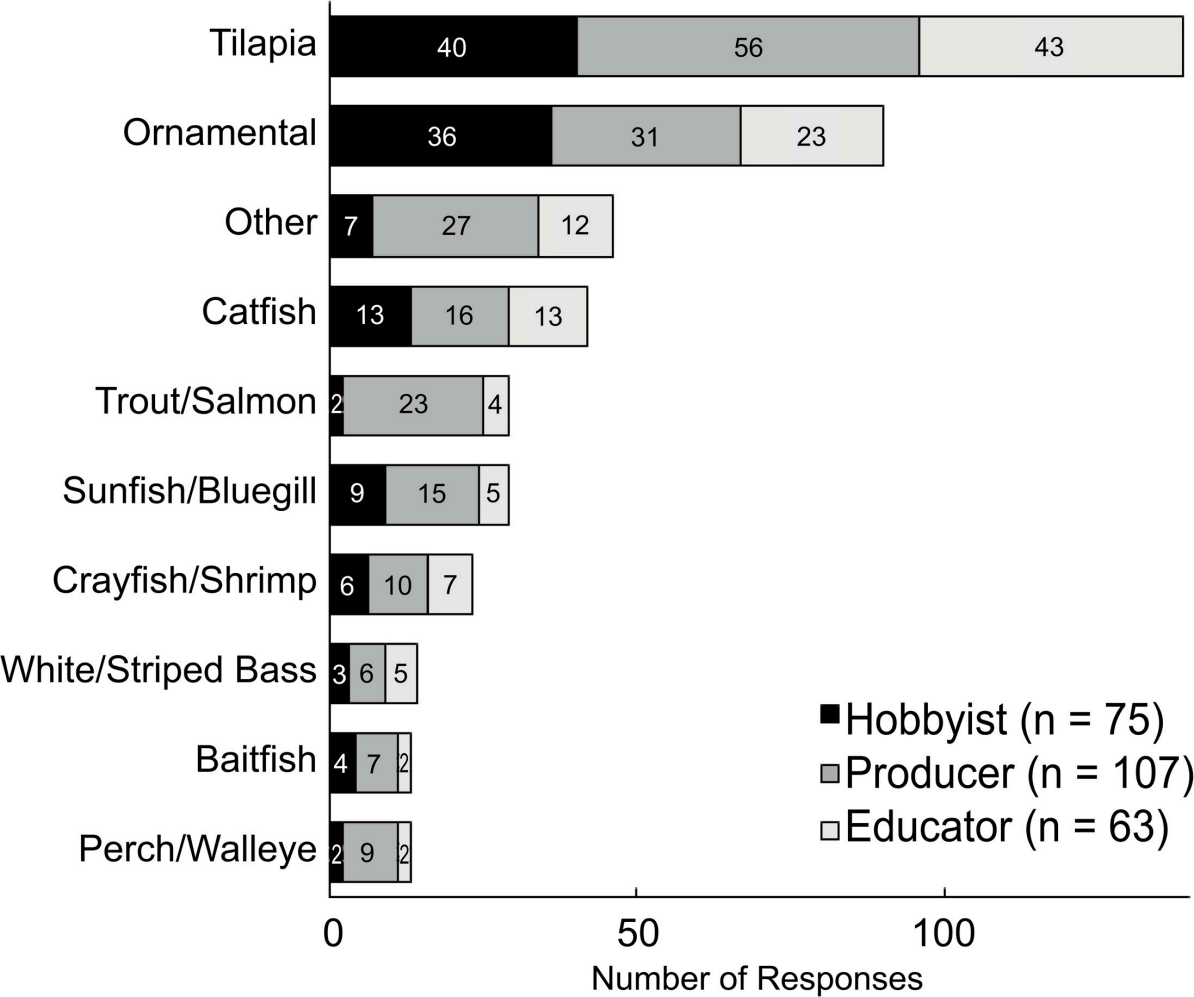
Frequency of fish species production by aquaponic hobbyists, producers, and educators. Numbers present within the bars represent the total number of selections made by each stakeholder group. Note–“Other” fish species grown included white seabass, paddlefish, northern pike, crappie, pumpkinseed sunfish, heat-tolerant tiger trout, arctic char, white sturgeon, sleepy cod, tenca, snakehead, Clarias catfish, and mummichog.

Respondents (N = 245) selected aquatic animals they produced from a list of common species grown in aquaponics (Fig 6). The average number of species grown per respondent was 1.6 ± 0.9 for hobbyists, 1.9 ± 1.3 for producers, and 1.9 ± 1.6 for educators, but not significantly different among groups (*p* = 0.386). Tilapia (*Cichlidae*) was by far the most commonly used fish species across all groups (57%), followed by ornamental fish (e.g. koi and goldfish; *Cyprinidae*) (37%), similar to Love et al. [6]. To a lesser extent “other” species, catfish (*Ictaluridae*), bluegill and other sunfishes (*Centrarchidae*), trout and salmon (*Salmonidae*), and crayfish, prawn, and shrimp (*Crustacea*) were grown. Very few respondents used striped bass (*Moronidae*), baitfish (*Cyprinidae*), perch and walleye (*Percidae*), largemouth bass (*Centrarchidae*), common or grass carp (*Cyprinidae*), barramundi (*Latidae*) or jade perch (*Terapontidae*). Of the great diversity of aquatic species being used experimentally by survey respondents (Fig 6), especially enticing is the use of saltwater shrimp. Marine aquaponics is relatively new and not thoroughly researched. Finding commercially valuable, salt-tolerant plant species can be challenging. Mariscal-Lagarda et al. [63] showed low salinity shrimp (*Litopenaeus vannamei*) could be incorporated with tomato production and Pinheiro et al. [64] used biofloc technology in the integration of saltwater shrimp and sea asparagus (*Sarcocornia ambigua*). Aquaponic researchers have also integrated marine fish production with a nursery facility Smooth Cordgrass (*Spartina alterniflora*) and Black Needlerush (*Juncus roemerianus*), for conservation and restoration of estuarine habitats [65, 66]. More research is needed in this area to determine viability. Fish production tends to operate at a break-even or financial loss in aquaponic operations [67]. Sale of non-food fish, particularly high-value ornamental species (e.g. koi), or longer-lived species that require long production periods (e.g. sturgeon) could be used on an industrial scale as an opportunity for aquaponics because of reduced sorting and harvesting costs. Alternative species that can be stocked at extremely high densities (e.g. *Clarias* catfish) may provide opportunities to maximize system profits by reducing initial infrastructure costs but could also increase operational costs and risks [56].

### Plant species produced

**Fig 7:**
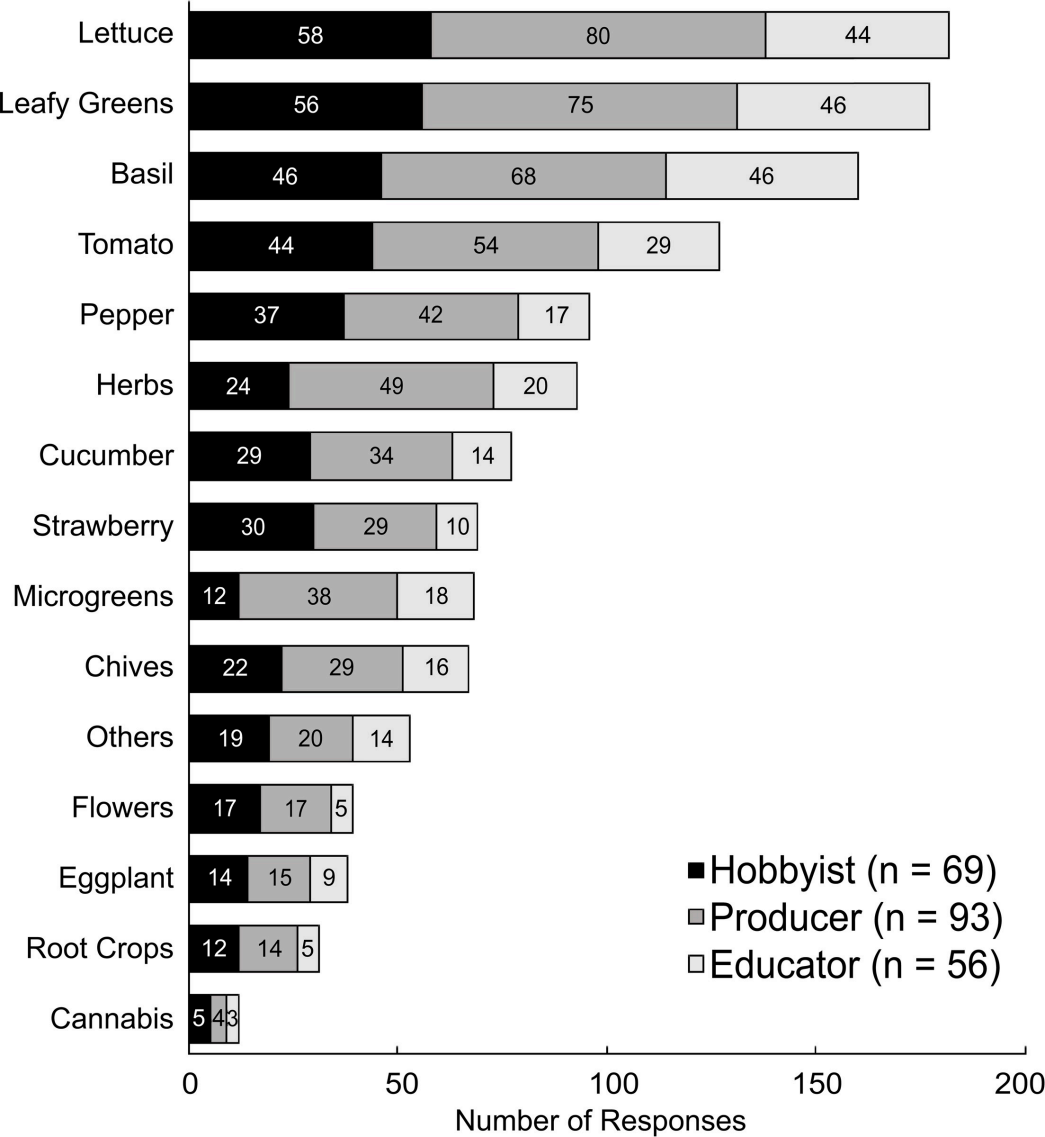
Frequency of plant species production by aquaponic hobbyists, producers, and educators. Numbers present within the bars represent the total number of selections made by each stakeholder group. Note—“Other” crops grown by participants included aloe, banana, bay tree, beans, bok/pak choi, brewer’s hops, broccoli, cauliflower, celery, cherry tomato, chili pepper, corn, cilantro, duckweed, edible flowers, ginger, green beans, kale, luffa, mango, medicinal herbs, melons, mint, Momordica charantia, okra, papaya, parsley, peanuts, peas, pineapple, pumpkins, raspberry, rosemary, squash, stevia, Swiss chard, turmeric, ulva, water lilies, watercress, and yam.

Respondents (N = 218) selected plant varieties produced from a list of common crops grown in aquaponics (Fig 7). The average number of crops grown per participant was 6.2 ± 2.9 for hobbyists, 6.1 ± 3.2 for producers, and 5.3 ± 2.4 for educators, which was not significantly different between groups (*p* = 0.177). This represents a lower plant diversity than the average of 8 ± 5 species reported by Love et al. [6]. The most commonly grown crops overall were lettuce (83%), leafy greens (81%), followed by basil (73%), tomatoes (58%), peppers (44%), and herbs (43%). Lesser-grown crops were cucumber (35%), strawberries (32%), microgreens (31%), chives (31%), “other” (24%), flowers (18%), eggplant (17%), root crops (14%), and cannabis (6%). Proportionally, the most common crops in this study were vegetative, with lower use of fruiting crops, and very low use of rooting crops. Lettuce and leafy greens were produced more commonly in this study than reported by Love et al. [6], whereas the proportion of growers producing herbs, tomato, pepper, and cucumber decreased. Lettuce, leafy greens, and herbs are relatively high-value, have short growth cycle varieties, are well suited to aquaponics and are very common in commercial production [35]. Love et al. [7] reported a higher use of leafy greens and lettuce by commercial producers compared to other stakeholders, although, proportional use of these crops was similar among stakeholder groups in this study. Fruiting crops like tomato, pepper, and cucumber are high value vegetable crops, but do not afford the grower the same value proposition in commercial production because of the lower turnover rate and per unit value compared to leafy greens and herbs [35, 52]. Few participants in this study indicated that they produced cannabis (6%), which is not surprising considering cannabis production has only recently been legalized in some U.S. States [17] and is still contentious and illegal in many states. Rooting crops are not well suited to aquaponic production and food safety concerns about the edible portion contacting the fish effluent water tend to discourage their use [58, 59]. A study by Dorick et al. [60] that used a surface water source found aquaponic water should be monitored more closely during June through January when *Escherichia coli* (*E*. *coli*) levels are highest. Additionally, once water leaves the fish culture unit it should be held in a storage tank for 8–16 days before use in crops to allow *E*. *coli* populations to fall below federally allowable limits [68] and reduce the likelihood of foodborne pathogens to contaminate produce [60].

## Conclusions

This study provides a comprehensive snapshot of the aquaponics industry. Academic institutions and Extension professionals can utilize information presented here to develop inclusive research and outreach initiatives that address bottlenecks to industry development. Likewise, stakeholder groups can use these results to gain insights into current production practices and avoid pitfalls commonly encountered at different stages of aquaponic implementation. The survey responses primarily reflect activities in the US in temperate and subtropical climates. Systems are mostly homemade/do-it-yourself, coupled, recirculating systems that incorporate deep-water culture or media beds. Common products include vegetable produce (leafy greens and vegetables), training and education, food fish (tilapia), ornamental fish (koi and goldfish), and microgreens. Sunlight and LED grow lights are most commonly used, along with municipal and well water. Labor inputs are minimal at the common system scales (< 3,000 ft^2^), but positively correlated with system size. Operations are often personally financed but grants and loans are available.

As the aquaponic industry matures, it is prudent to track advances made by stakeholders. As more information and technologies are made available to a wider audience, aquaponics practitioners are expanding on traditional models of success to include system design modifications, alternative fish and plant species, and increased facility size. Saltwater aquaponic systems are also on the rise, growing species like Penaeid shrimp. Respondents displayed a greater emphasis on commercial production, with less emphasis on education and hobbyist activities and 2.8x greater median production area reported in this study compared to those surveyed by Love et al. [6]. Successful aquaponic operations tend to be larger in scale, located in warmer climates, use aquaponics as a primary income source, obtaining higher product selling prices, have a gross revenue exceeding $5,000 annually, and sell non-food products like materials and supplies, training, agritourism, and consulting services [2, 7]. Commercial producers in this study tended to sell more types of products than other stakeholders, suggesting that diversification of offerings may be key to profitability. Diversification of fish and plant crops with emphasis on high value and low per unit production cost over time will be critical to profitability going forward.
